# Has Scots pine (*Pinus sylvestris*) co‐evolved with *Dothistroma septosporum* in Scotland? Evidence for spatial heterogeneity in the susceptibility of native provenances

**DOI:** 10.1111/eva.12395

**Published:** 2016-07-18

**Authors:** Annika Perry, Anna V. Brown, Stephen Cavers, Joan E. Cottrell, Richard A. Ennos

**Affiliations:** ^1^ Centre for Ecology and HydrologyPenicuikUK; ^2^ Forestry CommissionEdinburghUK; ^3^ Northern Research StationForest ResearchRoslinUK; ^4^ Institute of Evolutionary BiologyUniversity of EdinburghEdinburghUK

**Keywords:** adaptive trait, co‐evolution, Dothistroma needle blight, *Dothistroma septosporum*, host–parasite interactions, quantitative genetics, scots pine

## Abstract

Spatial heterogeneity in pathogen pressure leads to genetic variation in, and evolution of, disease‐related traits among host populations. In contrast, hosts are expected to be highly susceptible to exotic pathogens as there has been no evolution of defence responses. Host response to pathogens can therefore be an indicator of a novel or endemic pathosystem. Currently, the most significant threat to native British Scots pine (*Pinus sylvestris*) forests is Dothistroma needle blight (DNB) caused by the foliar pathogen *Dothistroma septosporum* which is presumed to be exotic. A progeny–provenance trial of 6‐year‐old Scots pine, comprising eight native provenances each with four families in six blocks, was translocated in April 2013 to a clear‐fell site in Galloway adjacent to a DNB‐infected forest. Susceptibility to *D. septosporum*, measured as DNB severity (estimated percentage nongreen current‐year needles), was assessed visually over 2 years (2013–2014 and 2014–2015; two assessments per year). There were highly significant differences in susceptibility among provenances but not among families for each annual assessment. Provenance mean susceptibility to *D. septosporum* was negatively and significantly associated with water‐related variables at site of origin, potentially due to the evolution of low susceptibility in the host in response to high historical pathogen pressure.

## 1 Introduction

Pathogens impose major selective pressure on tree species resulting in changes to host distribution, density and abundance (Hamilton et al., 2013). Where pathogens are endemic, and host and pathogen have coexisted for significant periods of time, it is expected that the genetic composition of both the host and pathogen will change through reciprocal co‐evolution (Ennos, 2015). If pathogen pressure varies substantially in time and space (Bulman, Gadgil, Kershaw, & Ray, 2004; Guernier, Hochberg, & Guegan, 2004; Watt, Palmer, & Bulman, 2011), tree populations in different parts of a species' range will show genetic variation for disease‐related traits (Hamilton et al., 2013) and spatial heterogeneity in disease susceptibility. In areas of high pathogen pressure, where there is strong selection for low host susceptibility it is expected that under common‐garden conditions, trees from these areas will show lower quantitative susceptibility to pathogens than those originating from areas of low pathogen pressure (Ennos, 2015).

In contrast, introduction of an exotic pathogen is expected to be devastating to naïve (and susceptible) hosts across their range. With no evolved defences, the spread of the pathogen will be restricted only by limiting abiotic conditions (Ennos, 2015). Thus, where pathogens are introduced, susceptibility in the host is likely to be uniformly high, and less related to variation in environmental factors that affect pathogen pressure across the geographic range of the host.

By studying trees from a range of populations exposed to pathogens under common environmental conditions, patterns of quantitative genetic variation for disease‐related traits can be quantified among and within these populations. Among‐population comparisons can be used to distinguish between situations involving endemic, co‐evolved pathogens, where variation in susceptibility to pathogens will be related to spatial patterns of pathogen pressure, and exotic pathogens, where susceptibility is likely to be uniformly high (Ennos, 2015). In order to verify reciprocal co‐evolution and to establish whether quantitative variation in host susceptibility has led to pathogen evolution, quantitative variation in an adaptive trait, such as aggressiveness, would also ideally be studied. Data on levels of genetic variation in susceptibility to pathogens within host populations can be used to determine the potential for future adaptation to the pathogen. Practical applications of this information can be broad and long term, although they may take significant time and resources to implement. Evidence of quantitative variation in susceptibility to pathogens can be used to establish breeding programmes selecting for this trait, as has been achieved for *Pinus taeda* for variation in susceptibility to fusiform rust (*Cronartium quercuum* f.sp. *fusiforme*; Carson & Carson, 1989; Eneback, Carey, & Flynn, 2004). In native populations, facilitating regeneration can allow high levels of pathogen pressure to act as a selective force (Burns, Schoettle, Jacobi, & Mahalovich, 2008) leading to rapid adaptation (Greene & Appel, 1994) in subsequent generations.

Currently, one of the most economically important diseases of pines worldwide (Barnes, Crous, Wingfield, & Wingfield, 2004) is Dothistroma needle blight (DNB) caused by the ascomycete fungal pathogen *Dothistroma septosporum* (and occasionally *Dothistroma pini*): more than 85 species are known to be affected (Brown, Stone, & Clayden, 2012) in every continent except Antarctica. Symptoms, including red‐brown lesions on needles, can lead to premature needle loss and, in severe cases, tree death. Development of DNB generally begins after bud flush in the spring when needles are infected by conidia. Lesions and then acervuli emerge gradually releasing further conidia within 12 weeks of initial infection (Gadgil, 1967) although this period may vary considerably depending on environmental conditions. In conditions favourable to the pathogen (high water availability, high humidity), a single cohort of needles may be subjected to multiple infection cycles (Bulman et al., 2004; Mullett, 2014). Premature shedding of infected needles is frequently observed (Gadgil, 1970). The origin of *D. septosporum* is not known although Central American cloud forests (Evans, 1984) and the Himalayas (Ivory, 1994) have both been proposed as centres of its natural distribution. There is also evidence that the disease may have been prevalent in British Columbia, Canada, for nearly 200 years, and it is therefore considered endemic to this region (Welsh, Lewis, & Woods, 2009).

There have been records of DNB in Britain for over 60 years (Murray & Batko, 1962), but in recent years, it has been reported with increasing frequency and severity (Brown & Webber, 2008) although this may partly be due to increased monitoring and surveillance programmes. Pine (predominantly Scots pine: *Pinus sylvestris*; lodgepole pine: *Pinus contorta*; Corsican pine: *Pinus nigra* ssp*. laricio*) accounts for around 15% of the total woodland resource in Great Britain (Brown et al., 2012), and DNB therefore poses a significant economic threat as these are important commercial timber species. Moreover, Scots pine is a key component of native Caledonian pinewood and many semi‐natural woodland types throughout Great Britain, so DNB is also a significant ecological and social threat. It is unknown whether *D. septosporum* is introduced, or is endemic and has co‐evolved with native *P. sylvestris*. In the case of the latter, quantitative variation in susceptibility of native populations would be related to historical environmental factors affecting *D. septosporum* growth, development and infection (Ennos, 2015): selection for lower susceptibility to *D. septosporum* would be expected in areas where the environment was most conducive to the pathogen and where the pathogen pressure was therefore high. Current DNB management strategies are focussed on making the environment suboptimal for the pathogen, including decreasing humidity through weed control and thinning, and removal of high inoculum‐producing hosts to reduce inoculum pressure (Forestry Commission Scotland 2013). Understanding the contribution of genetically controlled variation in susceptibility would potentially allow strategies to be developed, such as forestry breeding programmes for low susceptibility.

Despite significant fragmentation to <1% of its maximal area of occupation (Kinloch, Westfall, & Forrest, 1986; Mason, Hampson, & Edwards, 2004), 84 fragmented populations of native Scots pine forest remain in Scotland. Evidence based on genetic markers suggests these fragments have retained genetic variability and are highly connected by gene flow (Kinloch et al., 1986; Wachowiak, Iason, & Cavers, 2013; Wachowiak, Salmela, Ennos, Iason, & Cavers, 2011). Topography and the effect of oceanic currents contribute to significant spatial heterogeneity in the climate experienced by these native Scots pine populations (Salmela et al., 2010). Western sites experience more than three times as much rainfall and days with an average temperature above 5°C than eastern sites. Given the infection process in *D. septosporum* which requires leaf wetness, preferably continuously, under warm (day/night temperatures: 20/12°C) conditions (Gadgil, 1974), this will mean that western populations are likely to experience far higher pathogen pressure than eastern populations if *D. septosporum* is present. Therefore, in a co‐evolution scenario with an endemic pathogen, we would predict quantitative susceptibility to *D. septosporum* to be lower in western than in eastern populations. The aim of our study was to test this prediction by measuring quantitative variation in susceptibility to *D. septosporum* in native Scots pine populations from across their range in Scotland. We also analysed possible relationships between the quantitative variation in susceptibility of populations and environmental variables affecting probability of DNB infection historically present at their site of origin. Finally, we quantified the extent of genetic variation in susceptibility within populations to assess their potential for future adaptation to DNB.

Inoculating trees within a common environment allows variation in host response to be examined while minimizing the confounding effects of environmental variation and subjecting hosts to a common inoculum (Telford, Cavers, Ennos, & Cottrell, 2015). Although artificial conditions can best control spatial and temporal variation in the environment (including pathogens), natural inoculations are preferable when considering long‐term host responses and pathosystem dynamics in a fluctuating environment. The ability to extrapolate from the results of one method to the other may therefore be extremely useful. Fraser, Mullett, Woodward, and Brown (2015) have reported strong positive correlations between natural and artificial inoculation trials when infection levels were high. Results from a progeny–provenance artificial inoculation trial based on a single isolate by Perry et al. (2016) provide a suitable comparison with a progeny–provenance natural inoculation trial.

A natural inoculation trial was established in which levels of quantitative variation in susceptibility to *D. septosporum* were determined within and among provenances and maternal families of native Scots pine over multiple seasons and years. Differences in susceptibility to *D. septosporum* among provenances and families within provenances were measured, and narrow‐sense heritability and evolvability values were estimated. Under a scenario of endemic *D. septosporum* and co‐evolution with native Scots pine, our expectation was that provenances from the wetter conditions of western coastal Scotland would show lower susceptibility to *D. septosporum* than those from the drier eastern sites when subjected to natural inoculation under field conditions in a common garden trial. Our results, derived from natural inoculation by multiple *D. septosporum* isolates, were compared with those from a similarly structured trial carried out with a single isolate under glasshouse conditions ideal for disease development.

## 2 Methods

### 2.1 Source material

**Figure 1:**
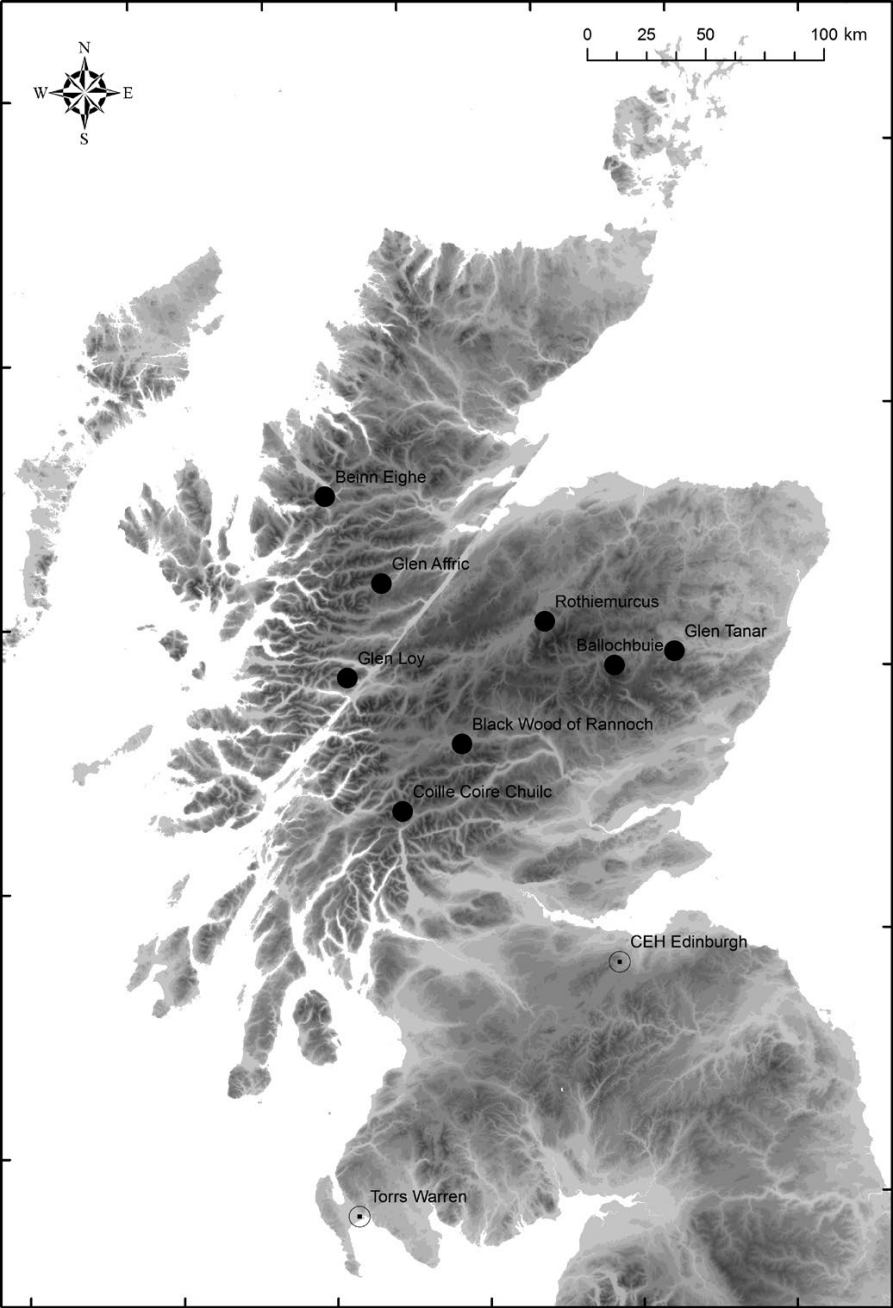
Location of each Scots pine provenance in Scotland. Provenances are indicated with black circles. Trees were grown in a common environment at CEH Edinburgh and were translocated to a naturally infected site in Torrs Warren forest, Galloway (both indicated with open circles)

At least 20 cones were collected from each of four open‐pollinated mother trees (minimum 100 m apart) in each of eight native Scottish Scots pine forests (Table 1, Fig. 1: BE, Beinn Eighe; BB, Ballochbuie; BW, Black Wood of Rannoch; CCC, Coille Coire Chuilc; GA, Glen Affric; GL, Glen Loy; GT, Glen Tanar; RM, Rothiemurcus) in March 2007. Seeds were extracted and sown on trays containing a 3:1 ratio of John Innes #1 compost: sand. Once the first needle whorls had emerged, six seedlings per family (putative half‐siblings) were transferred to 0.62‐L pots in June 2007. After bud flush in spring 2008, seedlings were transferred to 1.5‐L pots containing John Innes #3 compost. Seedlings were grown on in an unheated glasshouse at the Centre for Ecology and Hydrology (CEH) in Midlothian, Scotland (latitude 55.861, longitude −3.208) in ambient light until summer 2009, after which they were moved to external benching and watered as necessary. Trees were transferred to 6‐L pots containing John Innes #3 compost mixed with sand in March 2013.

**Table 1:** Collection and climatic information for each Scots pine provenance

Provenance	Code	Families	Lat^a^	Long^a^	ALT^a^	MRH^b^	AP^b^	ARD1^b^	CT^c^
Beinn Eighe	BE	21; 23; 26; 30	57.63	−5.40	48	81.78	1957.10	60.83	3.9
Glen Loy	GL	1868; 1872; 1876; 1877	56.91	−5.13	170	81.96	2951.56	64.46	4.1
Glen Affric	GA	1892; 1893; 1897; 1900	57.26	−4.92	268	81.82	2516.41	67.56	4.5
Coille Coire Chuilc	CCC	1801; 1806; 1807; 1809	56.42	−4.71	271	82.23	2681.17	68.83	5.0
Black Wood of Rannoch	BW	1822; 1825; 1828; 1830	56.68	−4.37	278	81.75	1445.75	54.60	5.7
Rothiemurcus	RM	1841; 1845; 1846; 1848	57.15	−3.77	314	81.32	986.60	48.93	5.8
Ballochbuie	BB	74; 75; 80; 97	56.98	−3.30	483	81.04	965.23	42.12	6.6
Glen Tanar	GT	1851; 1856; 1858; 1860	57.02	−2.86	334	81.48	800.36	39.19	6.3

Sources: ^a^hand‐held GPS during collection, ^b^UK Met. Office and ^c^Forestry Commission Ecological Site Classification.

Provenances are ordered within the table according to longitude (west to east).

Code, provenance codes; Lat, latitude; Long, longitude; ALT, altitude (m); MRH, mean relative humidity (%); AP, annual precipitation (mm); ARD1, annual rain days above 1 mm (%); CT, continentality.

### 2.2 Experimental design

Seedlings from four families from each of the eight provenances were laid out in a randomized block design, with six blocks containing one seedling per family per block (block *n* = 32, total *n* = 192). The design was established at CEH in June 2007, and the spatial arrangement was subsequently maintained throughout the duration of the trial. In April 2013, the 6‐year‐old trees were relocated to Torrs Warren forest in Galloway, Scotland (Fig. 1: latitude 54.864, longitude −4.888). They were laid out in their pots in a cleared area *c. *10 m north of a stand of >15 year‐old Corsican and lodgepole pine naturally infected with *D. septosporum*. Pots were spaced at 0.5 m intervals in a total area of 5.5 × 7.5 m^2^ with a guard row of pots containing single Scots pine trees on the outer edge at the same spacing to minimize edge effects. Pots were staked to the ground to maintain them in an upright position. During the experiment, 13 of the 192 trees died or were removed without permission.

### 2.3 Assessments

Infection in the cohort of current‐year needles was measured in every individual in the trial at two time periods (autumn and spring): the autumn assessment captured the preceding spring/summer period of infection while the spring assessment captured additional infection which occurred during the autumn/winter.

Estimated infection was recorded over 2 years (2013–2014 and 2014–2015), visually by the same observer at each time‐point, at approximately 6‐month intervals: September 2013; March 2014; October 2014; March 2015). The four assessments were carried out to establish the extent to which infection varied between seasons and years and to determine the consistency of relative infection levels for individual trees throughout the duration of the trial. To estimate susceptibility to *D. septosporum* for each tree, the percentage of total current‐year needles with DNB lesions (DNB severity) was assessed by applying a scale divided into 5% intervals. A score of 1% was used to indicate negligible levels of infection. Estimates obtained from autumn assessments in both years (2013–2014 and 2014–2015) are discussed as DNB severity resulting from spring/summer infection. Estimates recorded from spring assessments are discussed as total annual DNB severity as they reflect the cumulative symptoms from infection throughout the year. To derive an estimate of the percentage of infection which each year's needles acquired during the autumn/winter, DNB severity from the spring/summer was subtracted from total DNB severity estimates. Where these derived estimates are negative or zero, it is assumed that there was no infection in the autumn/winter, and that any reduction in DNB severity from estimates of infection from spring/summer is due to the loss of infected needles. DNB incidence at each assessment was recorded as the presence or absence of symptomatic needles on individual trees. A morphological trait (height at end of 2012, prior to translocation) was also measured so that the extent of variation, heritability and evolvability could be compared between the height and susceptibility to *D. septosporum* traits.

### 2.4 Climatic variables

#### 2.4.1 Environmental determination of pathogen pressure at trial site

Mean temperature and rainfall days (total number of days during with >1 mm rain) records for West Freugh, the nearest weather station to Torrs Warren (latitude 54.859, longitude −4.936; approximately 3.2 km from the trial), were used to assess the extent to which environmental variables influenced levels of susceptibility to *D. septosporum* during the trial. The association between environmental variables (mean temperature and percentage of rainfall days) in the 3 months prior to each assessment and mean DNB severity (log‐transformed) for each assessment was investigated. A period of 3 months was chosen as it is reported as the time taken for the full life cycle of *D. septosporum* to occur, from initial infection to lesion development and release of conidia in New Zealand (Bulman et al., 2004; Gadgil, 1967).

#### 2.4.2 Testing for adaptation to different pathogen pressures using climatic proxies

Both low temperature and low water availability are known to be major limiting factors for the life cycle of *D. septosporum* (Gadgil, 1974) although in a temperate climate the latter is thought to be more important (Watt, Kriticos, Alcaraz, Brown, & Leriche, 2009). UK Met. Office records (1971–2000 long‐term average data extrapolated from 5 km grid squares) for the site of origin for each provenance of Scots pine were used to select a range of proxy measures for water‐related variables (Table 1). Mean annual precipitation (AP, mm) and mean annual percentage of days during which there was >1 mm rain (ARD1, %) were chosen as indicators of the relative wetness of each site and the proportion of days during which the needles would be wet, respectively. A similar approach based on the amount and frequency of rainfall is also used by foresters in New Zealand to predict severity of DNB infection in *Pinus radiata* (Bulman et al., 2004). High levels of humidity are known to facilitate infection (Dvorak, Drapela, & Jankovsky, 2012). Therefore, we also included the mean annual relative humidity (MRH, %) as an additional environmental factor for consideration. A negative relationship between these variables at the site of origin and DNB severity at a ‘common site’ was expected if Scots pine had evolved in response to *D. septosporum* pathogen pressure over a long period of time.

Continentality (CT, values obtained from the Forestry Commission Ecological Site Classification; Pyatt, Ray, & Fletcher, 2001) is a qualitative measure of the effect of large bodies of water on land and is characterized by variation in multiple climatic variables, including temperature and precipitation. Where continentality is lower, fluctuations in temperature are reduced and the loss of water from needle surfaces as a result of evaporation is also expected to reduce. Provenances from areas which experience low continentality are expected to have been exposed to a higher historical pathogen pressure than provenances from regions of high continentality, and therefore, a positive relationship between continentality and mean annual provenance DNB severity was expected.

### 2.5 Statistical analysis

Prior to analysis, DNB severity was log‐transformed in order to normalize the distribution of residuals and to ensure equality of variances among families and provenances. Nested analyses of variance (ANOVA) were performed in Minitab 17 (Minitab Statistical Software, 2010) with provenance as a fixed effect, families nested within provenance as a random effect, and block as a random effect. Response variables were DNB severity (log‐transformed) or total height.

To assess the strength of the relationship between climate at the site of provenance origin and susceptibility to *D. septosporum* in the common garden trial, linear regressions were performed in R (R Core Team 2013) using provenance mean total DNB severity (log) and climatic variables previously described (Table 1).

Narrow‐sense heritability (*h* ^2^), which is the proportion of total phenotypic variance (*V*_P_) explained by additive genetic effects (*V*_A_; Falconer & Mackay, 1996), was estimated using among family, block and residual variance (*V*_fam_, *V*_block_ and *V*_res,_ respectively) from data pooled across provenances:

$$h^{2}=\frac{V_{A}}{V_{P}}=\frac{RV_{\mathrm{fam}}}{V_{\mathrm{fam}}+V_{\mathrm{block}}+V_{\mathrm{res}}}$$

where *R* is the relatedness of individuals within families. As the proportion of full to half‐siblings in each family was not known the following three relatedness scenarios were used: trees within a family are all half‐siblings (i.e. only share a ‘mother’; *R* = 4); trees within a family are 50% half‐ and 50% full siblings (*R* = 3); trees within a family are all full siblings (*R* = 2).

Standard errors for heritability estimates were calculated as follows (Vissher, 1998):

$$SE_{h^{2}}=R\sqrt{\frac{2{\left(1-\frac{h^{2}}{R}\right)}^{2}{\left[1+\left(s-1\right)\frac{h^{2}}{R}\right]}^{2}}{s(s-1)(f-1)}}$$

where *R* is the relatedness of trees within families, *s* is the mean number of offspring per family, and *f* is the mean number of families.

The coefficient of variation (CV_A_) is a standardized measure of variation normalized by the trait mean and provides a measure of the evolvability of a trait (Houle 1992). It was estimated for each trait as:

$${\text{CV}}_{A}=\frac{\sqrt{V_{A}}}{\mu_{\mathrm{trait}}}\times 100$$

where μ_trait_ is the mean of the trait of interest.

Results obtained from monitoring infection in a host species which has been transplanted into a natural environment can be very different to those from obtained from artificial inoculation experiments (Laine, 2006). This is due to variation in the pathogen and in the environment which are controllable in artificial conditions but can be highly spatially and temporally variable in natural experiments. A parallel study (Perry et al., 2016) has examined responses in native Scots pine progeny and provenances to DNB under artificial controlled conditions: the results of the current study were directly compared to test for consistency of response. Key differences and similarities between the two trials are described in Table S1. Although trees from common provenances were used, the provenances did not consist of the same families in both trials and therefore comparison between the trials is done on the basis of provenance means. Variation in DNB severity in the artificial inoculation trial was normally distributed, and the data were therefore not log‐transformed prior to analysis. To assess the comparability of artificial inoculation with natural inoculation trials (see Table S1 for descriptive list of differences), the responses of Scots pine provenances common to both trials were correlated. Pearson's correlation coefficients and significance values between mean provenance DNB severity following artificial inoculation (Perry et al., 2016) and following natural inoculation over 2 years were estimated using R. Consistency in DNB severity among seasons within and between each year was also assessed using Pearson's correlation coefficients and significance values.

## 3 Results

### 3.1 Environmental determination of DNB pressure and variation in DNB severity

Symptoms of DNB were found in the trial at every assessment. DNB incidence (percentage of trees with symptoms) across all trees in the trial ranged from 53.26% (spring/summer 2013) to 96.11% (spring/summer 2014), and every tree in the trial was symptomatic by the end of the 2014–2015 growing season.

There was a large amount of variation in susceptibility to *D. septosporum* within and among families, provenances, seasons and years (Table S2). DNB severity for all trees was log‐normally distributed at each time period. During both sampling years, mean DNB severity across all trees was low following the spring/summer infection period (2013–2014: 2.33 ± *SE* 0.31%; 2014–2015: 8.44 ± 0.89%). Although it increased during the autumn/winter (2013–2014: 11.35 ± 1.14%; 2014–2015: 11.11 ± 1.04%), mean disease levels remained low throughout the trial. Total mean DNB severity, accumulated by a single current cohort of needles across all trees, was also lower in 2013–2014 (13.01 ± 1.15%) than in 2014–2015 (18.23 ± 1.30%).

Mean DNB severity for each provenance was strongly positively associated with DNB incidence at each time period (Table S3), although by the second assessment period in 2014–2015, every tree was symptomatic. The correlation coefficient was statistically significant for infection which developed in the spring/summer and autumn/winter of 2013–2014 and the autumn/winter in 2014–2015.

Higher severity of DNB in 2014–2015 coincided with a greater number of rain days in the 3 months prior to assessments (Table S4) at the natural inoculation trial site. Although this suggests an association between the two, no formal relationship could be established due to the limited number of assessments. There was no clear relationship between temperature in the 3 months preceding assessments and DNB severity.

### 3.2 Variation among provenances in disease severity in relation to climate

**Figure 2:**
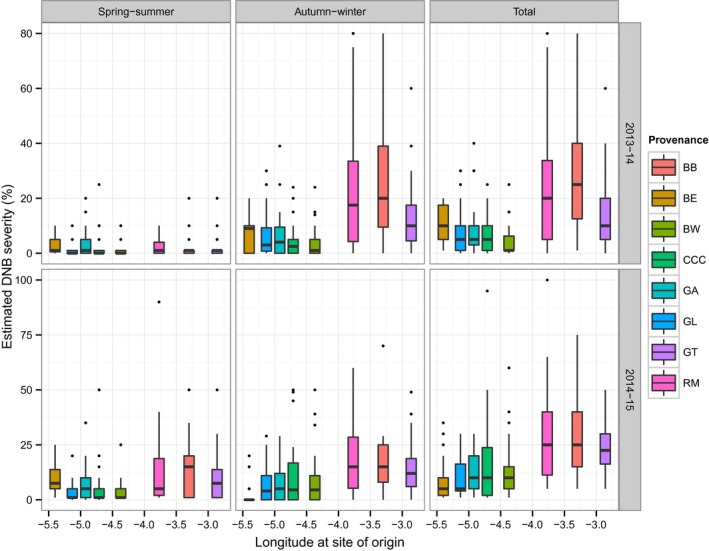
Variation in DNB severity within provenances for each season (spring/summer, autumn/winter, total) for each year (2013–2014, 2014–2015). Provenance codes are described in Table 1. Solid black lines indicate the median. The bottom and top of boxes indicate the first and third quartiles. The upper and lower whiskers extend to the highest and lowest values within 1.5 times the interquartile range. Individual points indicate outliers

There were highly significant differences among provenances in terms of susceptibility to *D. septosporum* in both years (ANOVA 2013–2014: *F*_7,24_ = 6.40, *p *<* *.001; ANOVA 2014–2015: *F*_7,24_ = 6.01, *p *<* *.001). The variation in, and levels of, susceptibility to *D. septosporum* within provenances was generally higher in provenances from the east of Scotland (BB, GT, and RM) than those from west or central Scotland (BE, BW, CCC, GA, and GL; Fig. 2) at every assessment except the first in 2013–2014. The three eastern provenances were ranked in the top three for susceptibility to *D. septosporum* at each assessment with the exception of the first assessment in 2013–2014 (Table S2). There was more variation in the order of ranking in the western and central provenances among seasons, and only GL maintained its ranking consistently in the bottom three for susceptibility to *D. septosporum*.

**Figure 3:**
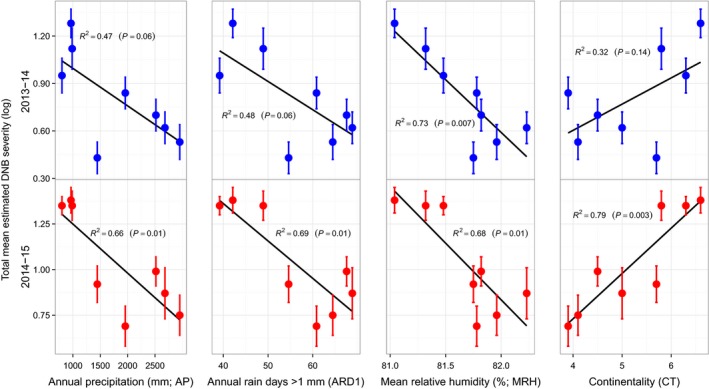
Linear regressions of climatic variables (as described in Table 1) and mean estimated DNB severity. Climatic variables are expected to affect pathogen pressure at the site of origin of each provenance. Total mean provenance DNB severity (log‐transformed) is given for each year (2013–2014: blue; 2014–2015: red). *R* ^2^ and significant (*p*) values are indicated for each regression.

The extent to which provenances from environments which are more favourable to the *D. septosporum* life cycle have adapted to a greater disease pressure through evolution in response to selection for low susceptibility to *D. septosporum* was examined (Fig. 3). Provenance mean DNB severity following 2 years of exposure (2014–2015) to *D. septosporum* showed significant (*p *<* *.01) negative regressions on measures of water availability (AP, ARD1, and MRH) at site of origin and a significant (*p *<* *.001) positive regression on continentality (CT) as hypothesized (Fig. 3). Significant regressions for disease severity in 2014–2015 on climatic variables associated with water availability explained between 66% and 79% of variation in susceptibility to *D. septosporum* among provenances: provenances with lower water availability at their site of origin were more susceptible to *D. septosporum*. There was a significant (*p *<* *.01) negative regression of provenance mean susceptibility to *D. septosporum* following 1 year of exposure to *D. septosporum* on mean relative humidity at the site of origin (Fig. 3), explaining 73% of variation in susceptibility to *D. septosporum* among provenances.

### 3.3 Heritability of variation in DNB disease severity and evolvability of variation

Estimated narrow‐sense heritability (*h* ^2^) of the trait susceptibility to *D. septosporum* varied depending on the assumed relatedness within families and on the year of assessment (Table S5). When trees within families were assumed to be full siblings, *h* ^2^ ranged from .11 to .34 across both years. These estimates doubled if trees within families were assumed to be half‐siblings. Highest *h* ^2^ estimates were obtained for annual DNB severity in 2014–2015, which also had higher mean DNB severity. Narrow‐sense heritability estimates for the height trait were measured for comparison and were around twofold higher than those estimated for susceptibility to *D. septosporum*. The genetic coefficient of variation (CV_A_), a measure of the evolvability of a trait, was highest for total DNB severity in 2014–2015 and was higher for susceptibility to *D. septosporum* than it was for height.

### 3.4 Comparison of naturally inoculated versus artificially inoculated trees

Estimated levels of susceptibility to *D. septosporum* in naturally inoculated trees and in artificially inoculated trees in semi‐controlled conditions were compared.

Mean DNB severity was between twofold and 19‐fold greater in the artificial inoculation trial (45.5 ± *SE* 1.8%) compared to DNB severity in this natural inoculation trial at each assessment. Despite large differences in the environmental conditions, the magnitude of the trees' response, the presence of multiple pathotypes in the naturally inoculated trial and the low number of provenances tested, there were consistent and strong (*r* > .63) positive associations between the two trials at each time period (Table 2; Fig. S1). Although the association between mean DNB severity in the artificial inoculation trial and in the time period in 2014–2015 of the natural inoculation trial is significant (*p *=* *.04), the lack of significance in other associations is likely to be a result of the low number of provenances tested.

**Table 2:** Correlation coefficients (*r*) and their associated significance (*p* value) for comparison of mean DNB severity among artificial and natural inoculation trials

Year	Spring/summer		Autumn/winter		Total	
	*r*	*p* value	*r*	*p* value	*r*	*p* value
2013–2014	.80	.10	.73	.16	.87	.06
2014–2015	.89	.04	.63	.25	.81	.10

DNB severity (%) following artificial inoculation was from one time‐point. DNB severity (log) following natural inoculation was estimated for each season (spring/summer and autumn/winter) and total DNB severity, for each year (2013–2014 and 2014–2015). Degrees of freedom (*df*) for all correlations was *N* − 2 (*df* = 3).

Those provenances which were least susceptible to *D. septosporum* in the natural inoculation trial (GL and BW) were also least susceptible in the artificial inoculation trial. The same is true of the highly susceptible provenances RM and GT in both trials. An exception appears to be GA, which has lower relative susceptibility in the natural inoculation trial compared to the artificial inoculation trial.

### 3.5 Consistency of susceptibility to DNB across seasons and years

Individual trees exhibited relatively high consistency in susceptibility to *D. septosporum* between seasons within a given year and between years. Correlations between seasons (Table S7) were all significant, with the exception of the first season in 2013–2014 with the total from each year (there were very low levels of DNB severity in the former) and between the first and second seasons in 2014–2015. There was little difference in DNB severity (≤20%) between seasonal and annual assessments in the majority of trees (>80% of trees, Table S6) with high consistency (≤5% difference in DNB severity between assessments) for around half of the trees in the trial in both seasonal and annual assessments. There was little consistency however in the rank order of DNB severity among families, or among western provenances over the 2 years (Table S2). The majority of trees had lower levels of estimated DNB severity following infection during the spring/summer compared to infection which developed during autumn/winter in both years (Table S2). The majority of trees had higher levels of DNB severity in 2014–2015 as compared to 2013–2014.

## 4 Discussion

Previous experimental studies on DNB on native Scots pine have been performed both in artificial conditions using a single pathotype in an ideal environment for infection (Fraser, Brown, & Woodward, 2015; Perry et al., 2016) and in a natural environment where DNB is known to affect neighbouring trees (Fraser, Mullett et al., 2015). High levels of variation in susceptibility to *D. septosporum* have been reported in all trials and significant differences among provenances were reported by Fraser, Brown et al. (2015) and Fraser, Mullett et al. (2015), although the composition of their trials did not allow a heritability estimate to be derived. Perry et al. (2016) found high heritability and significant differences in susceptibility to *D. septosporum* among families but not among provenances in an artificially inoculated trial.

In the current trial, native Scots pine trees, which have been naturally inoculated in the field with a high diversity (based on a panel of 11 microsatellites and mating‐type specific markers) of *D. septosporum* genotypes (Fraser, Mullett et al., 2015), show a large amount of variation in their levels of susceptibility to *D. septosporum* of which a significant proportion is heritable. Variation in susceptibility to *D. septosporum* is also significantly different among the provenances of Scots pine. This finding contrasts with that from a trial of similar size and design, which was artificially inoculated with a single isolate in conditions ideal for infection (Perry et al., 2016), where significant differences in susceptibility to *D. septosporum* were found among families but not among provenances. It is probable that differences between the trials in the proportion of variation in susceptibility to *D. septosporum* due to family and provenance reflect the different environments in which the disease developed. Extremely high levels of pathogen challenge in the artificial inoculation trial may also have masked differences in the response among provenances that were evident in the natural environment. Another possibility is that variation in pathogen aggressiveness led to variation in host response.

Observed variation in susceptibility to *D. septosporum* among provenances in the natural inoculation trial reveals a significant relationship between the climate at the site of provenance origin (specifically, the extent of water availability) and relative susceptibility to *D. septosporum*. Climate can be used as a proxy to predict the pathogen pressure that a provenance has been exposed to: provenances from areas which experience high water availability are likely to have been exposed to high pathogen pressure (Gadgil & Bulman, 2008) as the environment is favourable for *D. septosporum* dispersal (Dvorak et al., 2012) and symptom development (Gadgil, 1977).

High levels of quantitative variation between provenances in susceptibility to pathogens, as is observed here in Scots pine to *D. septosporum*, can result from adaptation to high pathogen pressure (Geiger & Heun, 1989). Our results are therefore consistent with the hypothesis that native Scottish provenances of Scots pine may have evolved with *D. septosporum* and that variation in susceptibility to *D. septosporum* reflects variation in the selection pressure imposed by infection: those provenances from areas where, for environmental reasons, pathogen pressure is greater are less susceptible to DNB. A similar finding has been made for provenance variation in disease symptoms in common garden trials of Douglas fir (*Pseudotsuga menziesii*) naturally infected with Swiss needle‐cast (*Phaeocryptopus gaeumannii*), which is associated with rainfall at the site of provenance origin (McDermott & Robinson, 1989). Similarly, a latitudinal cline of variation in susceptibility of *Eucalyptus globulus* to *Mycosphaerella* leaf disease (MLD; caused by *Teratosphaeria* spp.) revealed strong associations between MLD damage and temperature at site of provenance origin in multiple common garden trials (Hamilton et al., 2013). Alternative explanations may be that western provenances were better adapted to conditions in Torrs Warren and they were therefore less stressed and consequently less susceptible to DNB than eastern provenances, or that co‐evolution was not between Scots pine and *D. septosporum*, but a different pathogen which exerts a similar response in the host. The relationship between susceptibility to *D. septosporum* and climate at the site of host origin may also have arisen indirectly, rather than as a direct result of selection for low susceptibility in the presence of high pathogen pressure. Climatic variation across the range of Scots pine may have led to physiological or phenological variation in provenances which then indirectly affect their susceptibility to *D. septosporum*. A possible example may be variation in the size of stomata caused by variation in water availability: trees with smaller stomata may be less susceptible to *D. septosporum* than trees with larger stomata, as is observed in western white pine (*Pinus monticola*) to white pine blister rust (*Cronartium ribicola*; Woo, Fins, McDonald, & Wiese, 2001). Variation in needle morphology has been found among families and populations of native British Scots pine, with the number of stomatal rows negatively associated with latitude (Donnelly, Cavers, Cottrell, & Ennos, 2016). Therefore, in order to establish whether the results from this study are indeed indicative of a reciprocal co‐evolutionary relationship between Scots pine and *D. septosporum,* evidence for evolution of aggressiveness in *D. septosporum* in response to variation in host susceptibility should also be investigated.

Despite the strong relationship between climate at the site of provenance origin and susceptibility to *D. septosporum* there are individuals and families within the more susceptible eastern provenances which have relatively low levels of susceptibility: if these provenances have not co‐evolved with *D. septosporum*, why do they show variation in susceptibility? The geographic mosaic theory (Thompson, 2005) proposes three components to co‐evolution: geographic selection mosaics form across the landscape as a result of fitness in the host (or pathogen) depending on the frequency and distribution of pathogen (or host) genotypes, both of which are affected by landscape heterogeneity (including climate); co‐evolutionary hot and cold spots develop as a result of reciprocal or nonreciprocal selection, respectively; and traits remix across the landscape due to gene flow, random genetic drift and extinction events. In the absence of gene flow, a heterogeneous geographic distribution of resistance genes would be expected to develop (Hamilton et al., 2013), while admixture of resistance genes would be expected in species without restricted gene flow. Scots pine pollen is known to disperse over long distances (Robledo‐Arnuncio & Gil, 2005) and gene flow between native forests in Scotland is thought to have remained high despite fragmentation (Kinloch et al., 1986; Wachowiak et al., 2011, 2013). High levels of variation in susceptibility to *D. septosporum* among native provenances of Scots pine may therefore reflect the combined effects of local adaptation of forests to climatic heterogeneity in Scotland as well as high gene flow among populations (Loveless & Hamrick, 1984).

The *D. septosporum* pathotypes found at the trial site are known to be highly diverse (Fraser, Mullett et al., 2015), and the environmental conditions experienced during the trial were also very variable; tree genotype × pathogen genotype × environment (G_T_ × G_P_ × E) interactions were therefore likely to be very high (Thompson, 2005). Results from this experiment are consequently ideally contrasted with those from an artificial inoculation trial in which a single pathogen isolate was used in a controlled environment (Perry et al., 2016). Artificially inoculating Scots pine with *D. septosporum* under ideal conditions for infection and minimizing the environmental variation during the lifetime of the trial (including variation in the pathogen) using a similar number of individuals, families and provenances resulted in narrow‐sense heritability estimates (*h* ^2^) of .38–.75 depending on the relatedness of individuals within families (Perry et al., 2016). These values of *h* ^2^ are much higher than those obtained in the naturally inoculated trial reported here. This is to be expected as *h* ^2^ values are generally lower in natural conditions as a result of greater G_T_ × G_P_ × E interactions; *h* ^2^ in forest trees is rarely found to be >.3 (Carson & Carson, 1989). Narrow‐sense heritability estimates of susceptibility to *D. septosporum* following natural inoculation were also expected to be relatively low as there were no significant differences found among families. Additionally, it is possible that maternal effects may have led to overestimations of heritability for this trait (Roach & Wulff, 1987). Despite this, *h* ^2^ estimates of susceptibility to *D. septosporum* from our naturally inoculated trees were within the range reported for this trait in naturally inoculated *P. radiata*: .18 (Devey et al., 2004), .2 (Jayawickrama, 2001), .24 (Carson, 1989), .29–.51 (Chambers et al., 2000), .3 (Wilcox, 1982) and .36 (Ivković et al., 2010). Evolvability (CV_A_) of the trait was lower in the artificial inoculation trial (23.47) than in this study, but this is also expected due to G_T_ × G_P_ × E in natural conditions, whereas the latter two can be minimized in an artificial environment.

Evidence for quantitative genetic variation in susceptibility to *D. septosporum* in native Scots pine, coupled with high levels of estimated heritability and evolvability in this trait, suggests that there is significant potential to incorporate this trait into breeding programmes in the future, as has been achieved for New Zealand for *Pinus radiata* to DNB (Carson & Carson, 1989). Furthermore, it is hoped that current management strategies within native pinewoods, which facilitate regeneration, will contribute to rapid adaptation for low susceptibility to *D. septosporum* in Scots pine which are under high levels of disease pressure.

Direct comparison of the relative susceptibility of common provenances that occur in both the natural and artificial inoculation trials (Perry et al., 2016) also revealed good agreement between the different trials, although relative susceptibility to *D. septosporum* in one provenance (Glen Affric) was higher in the artificial trial. Inconsistencies, such as Glen Affric, in the associations between natural and artificial trials may be a result of other factors, including the use of different families. Fraser, Brown et al. (2015) and Fraser, Mullett et al. (2015) have shown similarly high correlation between a highly infected naturally inoculated trial and artificial inoculated trials, although when there were very low levels of infection in their natural trial the correlation ceased. Additionally, Fraser, Brown et al. (2015) and Fraser, Mullett et al. (2015) found significant differences in susceptibility to *D. septosporum* among naturally inoculated provenances in two of three assessed years, as well as in their artificial inoculation trials, despite the use of different provenances and younger trees. Understanding whether results can be extrapolated to larger temporal and spatial scales, and comparing more provenances and families would therefore be valuable in determining the strength of the association between the two methods. Naturally inoculated trials represent real‐life conditions (e.g. high diversity of pathogens, temporal environmental variation) better than artificial inoculation trials, and may therefore be more informative although environmental variation can confound results. Long‐term trials are useful in determining the effect of disease on health and fitness of hosts (van der Pas, 1981), which are necessary for understanding the short‐ and long‐term impacts on plantation and native forests. The results from this study therefore support the use of both trials in parallel as a highly valuable approach to understanding the response of trees to disease.

Quantitative variation in susceptibility to pathogens can result from variation in the environment at temporal and spatial scales (Telford et al., 2015). Seasonal variation in disease severity across the trial was associated with relative wetness at the trial site in the period preceding assessments; this has also been reported for the *Dothistroma*–*Pinus* pathosystem by (Fraser, Mullett et al., 2015; Gadgil & Bulman, 2008; Watt et al., 2009). The finding that there is relatively high consistency in susceptibility to *D. septosporum* recorded for individual trees between seasons and years supports the use of large scale in situ surveys which capture susceptibility to *D. septosporum* at a single time‐point, although interpretation of short‐term data sets should be treated conservatively (Fraser, Mullett et al., 2015). Presence or absence surveys of DNB across Scotland are conducted from June to August, while sites known to be infected are intensively surveyed from October to February (Griffin, 2014). These assessments should therefore successfully capture the presence and the scale of DNB, with the intensive survey recording annual infection impacts if conducted early in the following year.

The question then remains: if *D. septosporum* is endemic to Great Britain and has co‐evolved over a long period of time with native Scots pine trees, why has its recorded presence on Scots pine increased in recent years? A very similar situation has been reported in British Colombia, Canada where, despite evidence that suggests *D. septosporum* may have been endemic in native lodgepole pine forests for nearly 200 years (Welsh et al., 2009), extensive and increased levels of mortality are currently being seen (Woods, 2003). Although there is some suggestion that increasing host availability through establishment of plantation forests may have contributed to the increasing frequency and severity of outbreaks (Woods, Coates, & Hamann, 2005), significant associations between outbreaks and above‐average precipitation (Welsh, Lewis, & Woods, 2014) suggest that climate change has had a dramatic impact on the pathosystem. Mean annual precipitation in Great Britain has increased since the 1960s, and is expected to increase further as the climate continues to warm (UK Met. Office 2011). There has also been significant planting of susceptible species (predominantly Scots pine, Corsican pine and lodgepole pine) throughout Great Britain (currently *c*. 400,000 ha; Brown et al., 2012) compared with the remaining native Scots pine, which has a much narrower geographic distribution and occupies less land area (*c*. 18,000 ha; Forestry Commission Scotland 1998). The situation in British Colombia therefore serves as an indicator of the potential devastation that may result within endemic pathosystems where the balance tips in favour of the pathogen as a result of a change in the environment. In both British Colombia and the United Kingdom, *D. septosporum* is known to be highly diverse and sexually reproducing (Dale, Lewis, & Murray, 2011; Mullett, 2014). An alternative possibility is therefore that the pathogen has evolved an increase in aggressiveness, or that highly aggressive strains have been inadvertently introduced to the country. There have been no studies to date on the comparative aggressiveness or virulence of *D. septosporum* strains from different countries or environments.

This study provides evidence for the evolution of low susceptibility in Scots pine, possibly in response to the presence of high historical levels of *D. septosporum*. This would imply that the pathogen has been a part of the native Caledonian pinewood ecosystem for a significant period of time. The key findings are as follows: (1) quantitative variation in susceptibility to *D. septosporum* is very high; (2) positive associations between DNB severity and climates promoting high pathogen pressure; and (3) a significant proportion of the variation in susceptibility to *D. septosporum* is heritable. Evidently, therefore, native Scots pine provenances have substantial adaptive capacity to respond to DNB. However, if the increasing levels of the disease within Great Britain result from shifts in the wider environment such as climate change and landscape alteration, existing defences may ultimately be overcome and care should be taken to tackle the disease on multiple fronts.

## Conflict of Interest

The authors declare no conflict of interest.

## Data Archiving

Data for this study are available at NERC's Environmental Information Data Centre at http://eidc.ceh.ac.uk/ with reference http://doi.org/10.5285/99e028cc-4c3c-490b-bade-b9ef062a16e8.
