# Supplementary figures and images for: Has Scots pine (Pinus sylvestris) co‐evolved with Dothistroma septosporum in Scotland? Evidence for spatial heterogeneity in the susceptibility of native provenances
Source: Evol Appl. 2016 Jul 18;9(8):982–93. doi: 10.1111/eva.12395 (PMC4999528; doi:10.1111/eva.12395)

2013-14

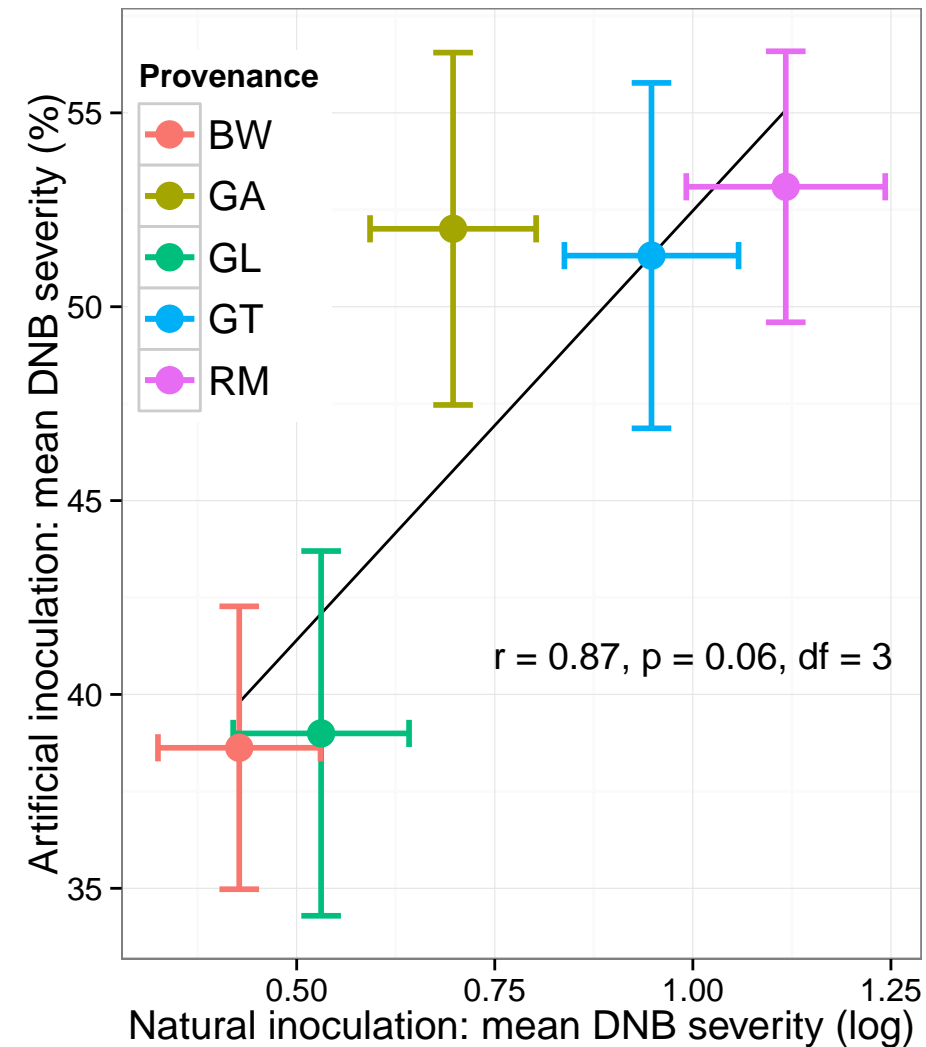

2014-15

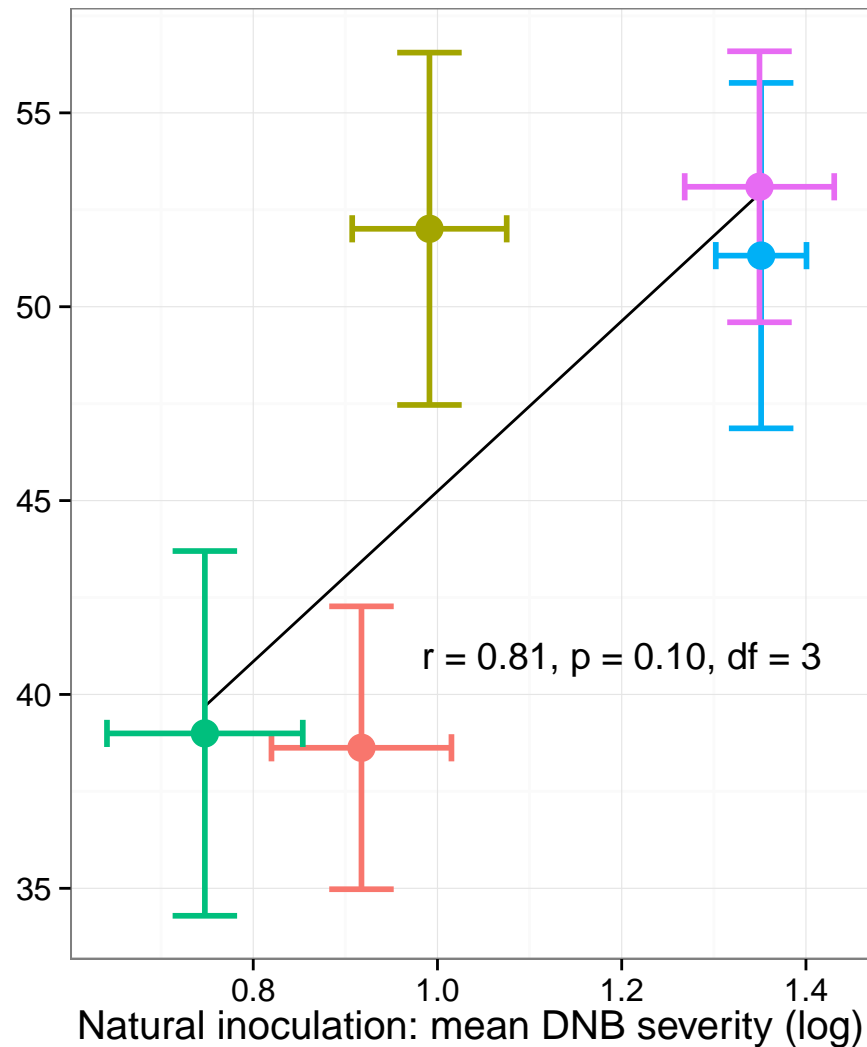

Supplement: Supplementary file 1 [file EVA-9-0982-s001.pdf]
