# Supplementary material for: Has Scots pine (Pinus sylvestris) co‐evolved with Dothistroma septosporum in Scotland? Evidence for spatial heterogeneity in the susceptibility of native provenances
Source: Evol Appl. 2016 Jul 18;9(8):982–93. doi: 10.1111/eva.12395 (PMC4999528; doi:10.1111/eva.12395)
Supplement: Supplementary file 2 [file EVA-9-0982-s002.docx]

**Supplementary materials**

Figure S1. Comparison of artificial and natural inoculation trials. Error bars are one standard error either side of the mean. DNB severity (%) for artificial inoculation is from a single detailed assessment. Total DNB severity for natural inoculation are from two years (2013-14 and 2014-15) and are log-transformed. Correlation coefficient (r), significance (p) and degrees of freedom (df) are provided for each year (Table 3).

Table S1. Mean estimated DNB severity (%) for each provenance and family and for all trees in the trial following each season (spring/summer; autumn/winter, total) over two years (2013-14 and 2014-15). Trial mean indicates the mean DNB severity over both years. Standard errors (SE) are given for each mean value. Number of trees (N) per provenance and family varies as a result of mortality or theft (no asterisk, N is constant in all assessments; *, N changes between 2013-14 and 2014-15 as indicated; **, N changes between seasons in 2014-15 as indicated). Rank order of DNB severity within provenances (N = 8) and families (N = 32) within each season is in parentheses (Rank 1 = highest DNB severity). Equal ranking is indicated (=). Provenances and families are ordered within the table according to longitude (west to east)

| Group | N | Mean estimated DNB severity (%) ± SE (rank order) | | | | | | | |
| --- | --- | --- | --- | --- | --- | --- | --- | --- | --- |
|  |  | 2013-14 | | |  | 2014-15 | | | Trial mean |
|  |  | Spring/summer | Autumn/winter | Total |  | Spring/summer | Autumn/winter | Total |  |
| *Trial* |  |  |  |  |  |  |  |  |  |
| All trees | 184, 180*, 179** | 2.33 ± 0.31 | 11.35 ± 1.14 | 13.01 ± 1.15 |  | 8.44 ± 0.89 | 11.11 ± 1.04 | 18.23 ± 1.30 | 10.72 ± 0.44 |
| *Provenances* | |  |  |  |  |  |  |  |  |
| BE | 23, 22* | 3.52 ± 0.76 (2) | 7.48 ± 1.37 (4) | 10.43 ± 1.49 (4) |  | 9.05 ± 1.46 (4) | 2.27 ± 1.13 (8) | 8.91 ± 2.11 (8) | 6.95 ± 0.63 (6) |
| GL | 24 | 1.04 ± 0.48 (7) | 6.42 ± 1.74 (6) | 6.88 ± 1.70 (6) |  | 3.38 ± 0.89 (8) | 6.79 ± 1.82 (7) | 9.83 ± 1.92 (7) | 5.72 ± 0.66 (8) |
| GA | 23 | 3.96 ± 1.15 (1) | 6.43 ± 1.99 (5) | 8.87 ± 2.20 (5) |  | 6.52 ± 1.74 (5) | 7.48 ± 1.81 (6) | 13.13 ± 1.74 (6) | 7.73 ± 0.76 (5) |
| CCC | 22 | 2.95 ± 1.29 (3) | 4.95 ± 1.45 (7) | 6.77 ± 1.49 (7) |  | 6.14 ± 2.38 (6) | 11.59 ± 3.49 (4) | 17.32 ± 4.82 (4) | 8.29 ± 1.2 (4) |
| BW | 24 | 0.96 ± 0.45 (8) | 4.04 ± 1.23 (8) | 5.00 ± 1.28 (8) |  | 4.00 ± 1.15 (7) | 10.08 ± 2.71 (5) | 13.67 ± 2.93 (5) | 6.29 ± 0.82 (7) |
| RM | 22 | 1.86 ± 0.58 (6) | 23.00 ± 5.11 (2) | 24.64 ± 5.20 (2) |  | 13.68 ± 4.32 (2) | 19.05 ± 3.95 (1) | 30.91 ± 5.17 (1) | 18.86 ± 1.93 (2) |
| BB | 23, 21*, 20** | 2.13 ± 0.95 (5) | 25.74 ± 4.27 (1) | 27.43 ± 4.14 (1) |  | 15.52 ± 3.29 (1) | 17.20 ± 3.42 (2) | 29.25 ± 3.89 (2) | 19.44 ± 1.63 (1) |
| GT | 23 | 2.30 ± 0.95 (4) | 13.48 ± 2.98 (3) | 14.91 ± 2.94 (3) |  | 10.55 ± 2.56 (3) | 15.59 ± 2.88 (3) | 25.23 ± 2.47 (3) | 13.60 ± 1.18 (3) |
| *Families* |  |  |  |  |  |  |  |  |  |
| BE21 | 6 | 1.67 ± 1.05 (=20) | 9.33 ± 1.86 (14) | 11.00 ± 2.58 (14) |  | 4.50 ± 1.36 (=23) | 0.00 ± 0.00 (32) | 3.17 ± 1.51 (32) | 4.94 ± 0.90 (28) |
| BE23 | 5 | 4.20 ± 1.77 (5) | 3.00 ± 2.00 (29) | 6.40 ± 2.71 (24) |  | 10.00 ± 2.74 (11) | 2.00 ± 1.22 (=31) | 11.00 ± 4.00 (24) | 6.10 ± 1.14 (25) |
| BE26 | 6 | 4.67 ± 1.80 (3) | 10.33 ± 2.63 (13) | 13.50 ± 3.20 (=13) |  | 8.67 ± 3.17 (13) | 2.50 ± 2.50 (29) | 8.67 ± 5.33 (29) | 8.06 ± 1.39 (=20) |
| BE30 | 6, 5* | 3.67 ± 1.54 (7) | 6.50 ± 3.59 (20) | 10.17 ± 3.32 (=17) |  | 14.00 ± 3.32 (8) | 5.00 ± 3.87 (=27) | 14.00 ± 4.85 (20) | 8.7 ± 1.48 (17) |
| GL1868 | 6 | 1.67 ± 1.05 (=20) | 3.50 ± 1.61 (=28) | 4.50 ± 1.36 (29) |  | 3.00 ± 0.89 (=28) | 6.17 ± 4.64 (23) | 9.17 ± 4.17 (28) | 4.67 ± 1.12 (29) |
| GL1872 | 6 | 2.17 ± 1.58 (14) | 0.33 ± 0.21 (32) | 0.83 ± 0.17 (32) |  | 2.83 ± 0.98 (29) | 2.67 ± 2.47 (28) | 4.83 ± 3.10 (31) | 2.28 ± 0.72 (32) |
| GL1876 | 6 | 0.17 ± 0.17 (=32) | 15.00 ± 4.55 (10) | 15.17 ± 4.55 (10) |  | 4.67 ± 3.15 (20) | 8.67 ± 3.59 (=20) | 13.33 ± 4.01 (=22) | 9.50 ± 1.68 (14) |
| GL1877 | 6 | 0.17 ± 0.17 (=32) | 6.83 ± 2.90 (18) | 7.00 ± 2.93 (=23) |  | 3.00 ± 1.57 (=28) | 9.67 ± 3.84 (18) | 12.00 ± 4.03 (23) | 6.44 ± 1.28 (24) |
| GA1892 | 5 | 3.00 ± 1.22 (10) | 7.20 ± 4.83 (16) | 10.20 ± 5.15 (15) |  | 11.00 ± 2.92 (10) | 5.00 ± 3.87 (=27) | 15.00 ± 3.54 (18) | 8.57 ± 1.60 (18) |
| GA1893 | 6 | 4.00 ± 3.20 (6) | 4.67 ± 2.33 (26) | 5.33 ± 2.46 (=28) |  | 4.5 ± 2.28 (=23) | 5.67 ± 2.85 (25) | 10.17 ± 3.32 (26) | 5.72 ± 1.10 (27) |
| GA1897 | 6 | 2.67 ± 1.67 (12) | 9.17 ± 6.17 (15) | 10.17 ± 6.14 (=17) |  | 7.33 ± 5.57 (16) | 8.67 ± 3.59 (=20) | 14.33 ± 3.84 (19) | 8.72 ± 1.90 (16) |
| GA1900 | 6 | 6.00 ± 2.65 (2) | 4.83 ± 2.24 (25) | 10.00 ± 4.08 (18) |  | 4.00 ± 1.90 (24) | 10.17 ± 4.58 (17) | 13.33 ± 3.80 (=22) | 8.06 ± 1.39 (=20) |
| CCC1801 | 6 | 1.83 ± 1.64 (=18) | 6.00 ± 3.92 (21) | 7.00 ± 3.91 (=23) |  | 0.67 ± 0.21 (32) | 16.33 ± 7.86 (7) | 17.00 ± 7.78 (15) | 8.14 ± 2.21 (19) |
| CCC1806 | 5 | 2.40 ± 1.91 (13) | 0.80 ± 0.80 (31) | 3.20 ± 1.93 (30) |  | 7.40 ± 3.56 (15) | 2.00 ± 1.22 (=31) | 8.60 ± 4.92 (30) | 4.07 ± 1.16 (31) |
| CCC1807 | 6 | 1.00 ± 0.82 (=26) | 6.67 ± 1.96 (19) | 7.67 ± 2.73 (21) |  | 11.17 ± 7.91 (9) | 12.33 ± 6.72 (13) | 23.50 ± 14.53 (10) | 10.39 ± 3.04 (12) |
| CCC1809 | 5 | 7.20 ± 4.83 (1) | 5.80 ± 3.69 (23) | 9.00 ± 2.92 (19) |  | 5.40 ± 2.56 (19) | 14.60 ± 9.36 (10) | 19.00 ± 8.57 (=13) | 10.17 ± 2.40 (13) |
| BW1822 | 6 | 0.67 ± 0.21 (27) | 5.00 ± 3.85 (24) | 5.67 ± 3.92 (26) |  | 6.17 ± 1.82 (17) | 11.33 ± 7.86 (15) | 16.67 ± 8.82 (16) | 7.58 ± 2.21 (23) |
| BW1825 | 6 | 0.33 ± 0.21 (30) | 5.83 ± 2.12 (22) | 6.17 ± 2.23 (25) |  | 5.67 ± 3.92 (18) | 6.00 ± 1.61 (24) | 10.83 ± 2.39 (25) | 5.81 ± 1.03 (26) |
| BW1828 | 6 | 1.00 ± 0.82 (=26) | 1.83 ± 0.87 (30) | 2.83 ± 1.62 (31) |  | 1.67 ± 0.67 (31) | 8.00 ± 5.68 (21) | 9.67 ± 5.53 (27) | 4.17 ± 1.39 (30) |
| BW1830 | 6 | 1.83 ± 1.64 (=18) | 3.50 ± 2.43 (=28) | 5.33 ± 2.46 (=28) |  | 2.50 ± 1.50 (30) | 15.00 ± 5.42 (9) | 17.50 ± 5.88 (14) | 7.61 ± 1.74 (22) |
| RM1841 | 6 | 2.00 ± 0.97 (=16) | 18.33 ± 12.76 (7) | 19.50 ± 12.58 (7) |  | 18.67 ± 14.33 (=4) | 13.83 ± 5.91 (12) | 32.50 ± 15.21 (=5) | 17.47 ± 4.60 (6) |
| RM1845 | 5 | 1.20 ± 0.97 (24) | 30.80 ± 12.42 (2) | 32.00 ± 11.79 (3) |  | 14.20 ± 3.83 (7) | 19.80 ± 6.00 (4) | 33.00 ± 4.64 (3) | 21.83 ± 3.61 (3) |
| RM1846 | 6 | 3.33 ± 1.67 (9) | 26.67 ± 9.19 (4) | 30.00 ± 10.41 (4) |  | 3.67 ± 0.84 (25) | 30.50 ± 9.36 (1) | 34.17 ± 9.08 (2) | 21.39 ± 3.66 (4) |
| RM1848 | 5 | 0.60 ± 0.24 (=29) | 16.40 ± 6.53 (8) | 17.00 ± 6.44 (9) |  | 19.20 ± 7.85 (2) | 10.80 ± 8.73 (16) | 23.00 ± 10.20 (11) | 14.50 ± 3.05 (9) |
| BB74 | 6, 5* | 2.00 ± 1.61 (=16) | 30.67 ± 8.57 (3) | 32.67 ± 8.61 (2) |  | 30.00 ± 5.70 (1) | 14.00 ± 5.10 (11) | 39.00 ± 4.30 (1) | 24.45 ± 3.30 (1) |
| BB75 | 6, 5** | 4.33 ± 3.23 (4) | 31.50 ± 11.90 (1) | 34.17 ± 10.83 (1) |  | 18.67 ± 7.44 (=4) | 17.60 ± 4.60 (6) | 30.00 ± 6.52 (7) | 22.65 ± 3.63 (2) |
| BB80 | 6, 5* | 1.33 ± 0.76 (=23) | 16.17 ± 5.61 (9) | 17.50 ± 5.59 (8) |  | 4.60 ± 2.20 (21) | 11.40 ± 2.48 (14) | 16.00 ± 2.92 (17) | 11.21 ± 1.84 (11) |
| BB97 | 5 | 0.60 ± 0.24 (=29) | 24.40 ± 6.75 (5) | 25.00 ± 6.89 (5) |  | 8.20 ± 2.87 (14) | 25.80 ± 11.82 (2) | 32.00 ± 12.21 (6) | 19.33 ± 3.63 (5) |
| GT1851 | 6 | 2.83 ± 1.62 (11) | 11.33 ± 4.05 (12) | 14.17 ± 3.52 (11) |  | 14.33 ± 7.62 (6) | 19.00 ± 5.60 (5) | 32.50 ± 3.82 (=5) | 15.69 ± 2.35 (8) |
| GT1856 | 6, 5* | 3.50 ± 3.30 (8) | 13.17 ± 3.92 (11) | 13.50 ± 3.91 (=13) |  | 3.60 ± 1.78 (26) | 20.40 ± 5.82 (3) | 24.00 ± 4.85 (9) | 12.76 ± 2.02 (10) |
| GT1858 | 6 | 1.33 ± 0.76 (=23) | 21.33 ± 9.54 (6) | 22.50 ± 9.46 (6) |  | 9.33 ± 1.86 (12) | 15.67 ± 7.08 (8) | 24.17 ± 6.25 (8) | 15.72 ± 2.90 (7) |
| GT1860 | 5 | 1.40 ± 0.93 (21) | 7.00 ± 3.18 (17) | 8.40 ± 3.87 (20) |  | 14.40 ± 5.98 (5) | 6.60 ± 2.34 (22) | 19.00 ± 3.32 (=13) | 9.47 ± 1.71 (15) |
|  |  |  |  |  |  |  |  |  |  |
|  |  |  |  |  |  |  |  |  |  |

Table S2. Number of rain days (> 1mm; percentage of total number of days), mean temperature (degrees Celsius) in the three months preceding each assessment and mean estimated DNB severity (%). Standard errors (SE) of mean estimated DNB severity are indicated.

| Year | Season | Mean DNB severity (%) ± SE | Days with > 1mm rain (%) | Mean temperature (°C) |
| --- | --- | --- | --- | --- |
| 2013-14 | Spring/summer | 2.33 ± 0.31 | 59.78 | 14.60 |
|  | Autumn/winter | 11.35 ± 1.14 | 95.56 | 6.20 |
| 2014-15 | Spring/summer | 8.44 ± 0.89 | 65.22 | 14.20 |
|  | Autum/winter | 11.11 ± 1.04 | 73.33 | 5.27 |

Table S3. Narrow sense heritability (*h^2^*), evolvability (*CV_A_*) and associated standard errors (SE) of the trait total DNB severity (log-transformed) in both years (2013-14 and 2014-15). A morphological trait, height (as measured at end of 2014 growth, mm), is provided for comparison. Heritability values are estimated for different assumptions of relatedness within families: R = 2, full siblings; R = 3, 50 % full siblings and 50 % half siblings; R = 4, half siblings.

| Trait | Proportion of variance due to: | |  | *h^2^* (SE) | | | CV_A_ |
| --- | --- | --- | --- | --- | --- | --- | --- |
|  | Family | Block |  | R = 2 | R = 3 | R = 4 |  |
| *Susceptibility to DNB* | | | | | | | |
| 2013-14 | 5.40 | 5.78 |  | 0.11 (0.37) | 0.16 (0.56) | 0.22 (0.74) | 34.37 |
| 2014-15 | 8.52 | 5.73 |  | 0.17 (0.41) | 0.26 (0.62) | 0.34 (0.82) | 30.00 |
| *Morphological trait* | | | | | | | |
| Height | 16.82 | 2.06 |  | 0.34 (0.47) | 0.50 (0.71) | 0.67 (0.95) | 7.96 |

Table S4. Comparison of natural and artificial ([Perry et al. 2016](#_ENREF_2)) inoculation trials.

| Variation in trials | Natural inoculation | Artificial inoculation |
| --- | --- | --- |
| Environment | Trees and pathogens exposed to daily and seasonal variation in weather | Conditions controlled to optimise infection: high water availability and warm temperature at all times |
|  |  |  |
| Pathogen | High diversity of *D. septosporum* ([Fraser et al. 2015](#_ENREF_1)) | A single isolate used to inoculate all trees |
|  |  |  |
| Tree provenance and families | Eight Scottish provenances  BB, BE, CCC  BW, GA, GL, GT, RM  No common families | Six Scottish provenances  SD  BW, GA, GL, GT, RM  No common families |
|  |  |  |
| Tree age at outset | Five years | Four years |
|  |  |  |
| Design | Progeny-provenance  Randomised block  Six blocks | Progeny-provenance  Randomised block  Seven blocks |
|  |  |  |
| Duration | Two years | Nine weeks |
|  |  |  |
| Assessment | Visual estimate of susceptibility to DNB | Destructive measure of susceptibility to DNB |

Table S5. Pairwise pearson correlation coefficient values (r) and associated significance (*, p < 0.05; **, p < 0.01; ***; p < 0.001) for estimated DNB severity of all individuals (N = 179-184) in each season (spring/summer, autumn/winter, total) in each year (2013-14, 2014-15).

| Year | Season | Correlation coefficient (r) | | | | | |
| --- | --- | --- | --- | --- | --- | --- | --- |
|  |  | 2013-14 | | |  | 2014-15 | |
|  |  | Spring/  summer | Autumn/  winter | Total |  | Spring/  summer | Autumn/  winter |
| 2013-14 | Autumn/winter | -0.20** |  |  |  |  |  |
|  | Total | 0.08 | 0.92*** |  |  |  |  |
| 2014-15 | Spring/summer | 0.19** | 0.23** | 0.34*** |  |  |  |
|  | Autumn/winter | -0.16* | 0.27*** | 0.22** |  | -0.14 |  |
|  | Total | -0.04 | 0.37*** | 0.39*** |  | 0.39*** | 0.78*** |

Table S6. Difference in recorded DNB severity between seasons (spring/summer compared to autumn/winter) and annually (total DNB severity in 2013-14 compared to 2014-15).

| Comparison of DNB severity | Trees (% of total) whose difference in DNB severity between assessments is: | | |
| --- | --- | --- | --- |
|  | ≤ 5 % | ≤ 10 % | ≤ 20 % |
| Seasonal: within 2013-14 | 50.54 | 70.65 | 84.78 |
| Seasonal: within 2014-15 | 45.81 | 64.80 | 81.01 |
| Annual: between 2013-14 and 2014-15 | 46.37 | 62.01 | 85.47 |

Table S7. Pearson’s correlation coefficient (r) and associated significance (p) for mean estimated DNB severity (log-transformed) and DNB incidence (per cent of trees with DNB symptoms) for each provenance at each season (spring/summer, autumn/winter, total) within each year (2013-14, 2014-15). Degrees of freedom for each correlation is six.

|  | Correlated DNB severity among seasons | | | | | | | |
| --- | --- | --- | --- | --- | --- | --- | --- | --- |
|  | Spring/summer | |  | Autumn/winter | |  | Total | |
|  | r | p |  | r | p |  | r | p |
| 2013-14 | 0.865 | 0.006 |  | 0.883 | 0.003 |  | 0.690 | 0.058 |
| 2014-15 | 0.649 | 0.082 |  | 0.967 | < 0.001 |  | - | - |

Fraser, S., M. S. Mullett, S. Woodward, and A. V. Brown. 2015. Between-site and -year variation in the relative susceptibility of native Scottish *Pinus sylvestris* populations to Dothistroma needle blight. *Plant Pathology* **Available online**:doi: 10.1111/ppa.12425.

Perry, A., W. Wachowiak, A. V. Brown, R. A. Ennos, J. E. Cottrell, and S. Cavers. 2016. Substantial heritable variation for susceptibility to *Dothistroma septosporum* within populations of native British Scots pine (*Pinus sylvestris*). *Plant Pathology* **Accepted**.
